# Periosteum progenitors could stimulate bone regeneration in aged murine bone defect model

**DOI:** 10.1111/jcmm.15891

**Published:** 2020-09-15

**Authors:** Han Xiao, Linfeng Wang, Tao Zhang, Can Chen, Huabin Chen, Shengcan Li, Jianzhong Hu, Hongbin Lu

**Affiliations:** ^1^ Department of Sports Medicine Xiangya Hospital Central South University Changsha China; ^2^ Key Laboratory of Organ Injury, Aging and Regenerative Medicine of Hunan Province Changsha China; ^3^ Xiangya Hospital‐International Chinese Musculoskeletal Research Society Sports Medicine Research Centre Changsha China; ^4^ Hunan Engineering Research Center of Sport and Health Changsha China; ^5^ Department of Spine Surgery Xiangya Hospital Central South University Changsha China

**Keywords:** aged mice, bone defect, bone regeneration, periosteum derived cells, Prx1^−^ MSC, Prx1^+^ MSC

## Abstract

Periosteal stem cells are critical for bone regeneration, while the numbers will decrease with age. This study focused on whether Prx1^+^ cell, a kind of periosteal stem cell, could stimulate bone regeneration in aged mice. Four weeks and 12 months old Prx1CreER‐GFP; Rosa26^tdTomato^ mice were used to reveal the degree of Prx1^+^ cells participating in the femoral fracture healing procedure. One week, 8 weeks, 12 and 24 months old Prx1CreER‐GFP mice were used to analyse the real‐time distribution of Prx1^+^ cells. Twelve months old C57BL/6 male mice (n = 96) were used to create the bone defect model and, respectively, received hydrogel, hydrogel with Prx1^−^ mesenchymal stem cells and hydrogel with Prx1^+^ cells. H&E staining, Synchrotron radiation‐microcomputed tomography and mechanical test were used to analyse the healing results. The results showed that tdTomato^+^ cells were involved in bone regeneration, especially in young mice. At the same time, GFP^+^ cells decreased significantly with age. The Prx1^+^ cells group could significantly improve bone regeneration in the murine bone defect model via directly differentiating into osteoblasts and had better osteogenic differentiation ability than Prx1^−^ mesenchymal stem cells. Our finding revealed that the quantity of Prx1^+^ cells might account for decreased bone regeneration ability in aged mice, and transplantation of Prx1^+^ cells could improve bone regeneration at the bone defect site.

## 1 INTRODUCTION

Bone is a kind of tissue with perfect regeneration and remodelling ability, which gives rise to considerable interest for regenerative therapeutic, while age would profoundly impact the bone regeneration ability.^1^, ^2^ Animal experiments in mice and clinical studies in humans showed decreased bone regeneration ability with age.^3^, ^4^, ^5^ Many reasons are accounting for this: reduced number of osteogenic stem cells, reduced proliferation, differentiation ability, and decreased systemic or local blood flow, but we still do not know which one dominates and how they interact.^6^

Bone regeneration occurs by two major ossification processes, endochondral ossification in which the skeletal element first develops as a cartilaginous template that is subsequently replaced by bone, and intramembranous ossification in which mesenchymal cells directly differentiate into bone‐forming osteoblasts. The ossification process does not require pre‐existing cartilage.^7^, ^8^ Both procedures require the replenishment of the osteogenic or chondrogenic progenitor cells that participate in bone or cartilage formation during normal development and under pathologic conditions, such as fracture healing.^9^, ^10^ In general, osteogenic progenitors distribute in various bone compartments along the bone's outer surface within the periosteum and the inner surface of bone within the endosteum.^11^, ^12^ Histological, periosteum is composed of at least two layers, outer fibrous layer and inner cambium layer.^13^ The outer fibrous layer mainly contains fibroblastic cells, while the inner cambium layer contains several types of cells, such as fibroblasts, mesenchymal stem cells, osteogenic or chondrogenic progenitors.^14^ Many works have attempted to investigate the periosteal stem cells and determine the cell types.^15^, ^16^, ^17^ However, the identity of periosteal stem cells remains unclear.

A previous study found that Prx1 was a paired‐related homeobox gene expressed in a subset of periosteal cells in the cambium layer surrounding the long bones and cartilage.^18^ These Prx1 positive cells (Prx1^+^ MSCs) have the potential of osteogenic and chondrogenic differentiation and are essential for limb development and bone regeneration.^18^, ^19^, ^20^ Duchamp de Lageneste et al found that Prx1^+^ MSCs can efficiently contribute to cartilage and bone regeneration, while periostin was essential for maintaining the Prx1^+^ MSCs pool.^21^ Therefore, we wondered that if the quantity of Prx1^+^ MSCs would decrease with age and affect bone regeneration ability in aged mice.

In this study, we verified the role of Prx1^+^ MSCs in the young murine bone fracture model and its distribution characterization with age. We isolated Prx1^+^ MSCs and Prx1 negative mesenchymal stem cells (Prx1^−^ MSCs) from murine periosteum and used them to enhance bone defect regeneration in aged murine bone defect model. We also compared the osteogenic ability and proliferation ability between Prx1^+^ MSCs and Prx1^−^ MSCs. We found that Prx1^+^ MSCs had better osteogenic ability than Prx1^−^ MSCs and could significantly improve bone regeneration in aged murine bone defect model.

## 2 MATERIAL AND METHOD

### 2.1 Animal

Prx1CreER‐GFP (Stock No. 029211) and Rosa26^tdTomato^ (Stock No. 007909) mice were purchased from Jackson Laboratory. All the mice were housed in our animal facility in our university with controlled temperature and light cycles (24°C and 12/12 light cycle). This animal study was reviewed and approved by our Institutional Animal Care and Use Committees (No. 201703222).

### 2.2 Lineage tracing analysis

To verify the specific distribution of Prx1^+^ MSC on the periosteum of the femoral shaft with ageing, 1 week, 8 weeks, 12 and 24 months old mice (n = 3 per group) were killed, and the femurs were harvested for immunofluorescence analysis. To investigate the degree of Prx1^+^ MSC participating in bone regeneration in young and aged mice, 4 weeks and 12 months old Prx1CreER‐GFP;Rosa26^tdTomato^ mice (n = 3 per group) were received intraperitoneal injections of 75 mg/kg bodyweight tamoxifen (Sigma‐Aldrich) for 5 days before closed left femoral bone fracture was made.^20^ At 2 weeks after surgery, the mice were killed, and the femurs were harvested for immunofluorescence.

### 2.3 Cells isolation

Periosteal mesenchymal stem cells were isolated from C57BL/6 background transgenic mice using a modified method based on previously literature.^21^, ^22^ Briefly, hindlimbs were disconnected from the trunk of 4 weeks old Prx1CreER‐GFP mice, and the entire attached soft tissues were removed from the bone. After the epiphysis of tibias and femurs on both sides was cut off, the bone marrow was flushed out with α‐MEM (Hyclone) and the endosteum was removed by a drill (0.6 mm in diameter). The diaphyses of tibias and femurs were excised to chips with scissors. Bone chips were digested by a complete medium (containing 10% FBS [Gibco] and 1% penicillin/streptomycin [Hyclone]) supplemented with 1.5 mg/mL collagenase II (Gibco) for 1.5 hours in a shaking incubator at 37°C. Enzyme‐treated bone chips suspended in complete medium were seeded into 25 cm^2^ culture flask and cultured at 37°C in a humidified atmosphere consisting of 5% CO_2_ in incubator.

### 2.4 Fluorescence‐activated cell sorting

Passage 3 periosteal cells were trypsinized, resuspended in PBS, 5% FBS and then incubated with APC anti‐mouse CD11b (Invitrogen), APC anti‐mouse CD34 (Biolegend), PE anti‐mouse CD90 (Biolegend) and APC‐Cy7 anti‐mouse SCA‐1 (BD Biosciences). After washing with PBS twice, Prx1^+^ MSCs (GPF+ cells) and Prx1^−^ MSCs (CD90^+^SCA‐1^+^EGPF^−^ CD34^−^CD11b^−^ cells) were sorted by FACS (BD Biosciences) for further experiment.

### 2.5 Osteogenic differentiation

Cells (1 × 10^5^ per well) are seeded into 0.1% gelatin‐coated 12‐well culture plate. After cells reached 60%‐70% confluence, the medium was replaced to OriCell™ C57BL/6 Mouse Mesenchymal Stem Cell Osteogenic Differentiation Medium (Cyagen Biosciences). The medium was refreshed twice a week. After 3 weeks of osteogenic induction, the cells were fixed in 4% paraformaldehyde for 30 minutes and stained with Alizarin Red for 5 minutes. To quantify the osteogenic differentiation, we used the 10% cetyl pyridinium chloride (Sigma‐Aldrich) to solubilize the stain for 20 minutes. Then, the OD values of solutions were measured at 560 nm. Besides, cell samples were collected after 7 days of osteogenic induction for ALP staining, ALP activity assay and qRT‐PCR.

### 2.6 ALP staining and activity

After 7 days of osteogenic induction, the cell culture supernatants were collected and centrifuged to remove cell debris, in which ALP activity was then determined using an ALP activity detection kit (Jiancheng Bioengineering) according to the manufacturer's instructions. The adherent cells were washed three times with PBS, fixed in 4% paraformaldehyde for 20 minutes and stained with ALP using BCIP/NBT Alkaline Phosphatase Color Development Kit (Beyotime Institute of Biotechnology).

### 2.7 Real‐time qPCR

Total RNA was extracted using the TRIzol reagent (Invitrogen), and cDNA was synthesized with a GoScript™ Reverse Transcription System (Promega) according to the manufacturer's instructions. PCR reactions were performed on ABI PRISM^®^ 7900HT System (Applied Biosystems) with GoTaq^®^ qPCR Master Mix (Promega). Expression data were uniformly normalized to β‐actin, and the relative expression was calculated using the $2^{‐\Delta \Delta C_{t}}$ method. The primer sequences employed in the current study were listed in Table 1.

**TABLE 1:** Oligonucleotides used in qRT‐PCR

Gene	Forward	Reverse
ALP	AGGGTGGACTACCTCTTAGGTC	AGGGTGGACTACCTCTTAGGTC
SP7	ATGGCGTCCTCTCTGCTTG	TGAAAGGTCAGCGTATGGCTT
BMP‐2	GGGACCCGCTGTCTTCTAGT	TCAACTCAAATTCGCTGAGGAC
β‐actin	GGAGATCACAGCTCTGGCT	GTCGATTGTCGTCCTGAGG

Abbreviation: qRT‐PCR, quantitative real‐time polymerase chain reaction.

### 2.8 Femoral defect model and cell transplantation

The surgical procedure was performed as previous report.^23^ Briefly, 12 months old male C57BL/6 mice were anaesthetized with pentobarbital, and the skin incision was made over the thigh. Then, the left femur surface was exposed, and a hole (0.8 mm in diameter) was drilled into one cortex without drilling into the opposite cortex in the middle shaft. For cell transplantation, every 1 × 10^6^ cells were embedded in 20 μL hydrogel using a Flexcell^®^ Thermacol^®^ Kit and 20 μL cell hydrogel mixed component was transplanted into the defect site. After surgery, mice were allowed to move freely. At 2 and 4 weeks after surgery, radiographic analysis, histological analysis and biomechanical tests were used to assess the bone regeneration.

### 2.9 Synchrotron radiation‐microcomputed tomography (SR‐μCT) analysis

At 2 and 4 weeks after surgery, the left femurs were harvested and fixed in 4% paraformaldehyde. The microstructure of newly formed bone in the bone defect area was evaluated by SR‐μCT based on previous report.^24^ Each sample was fixed in a table that allowed 180° rotation at the centre of the rotary stage during scanning. Then, we set beam energy, exposure time and sample‐to‐detector distance at 18.0 keV, 0.5 seconds and 5.0 cm. All the 720 radiographic projections were imaged by the CCD detector with a pixel size of 3.25 μm. At the same time, dark‐field and flat‐field images were also captured to reduce the ring artefact during reconstruction. After the projections were transformed into 8‐bit slices, the phase retrieval of projected images was performed by PITRE software written by BL13W1. According to the previous report, the bone was extracted from soft tissue using a fixed threshold segmentation after a median filter reduced noise. Morphological parameters of the newly formed bone at the defect site, such as bone volume to total volume ratio (BV/TV) and trabecular thickness (Tb·Th), were calculated.

### 2.10 Histology

After radiographic assay, fixed samples were decalcified in EDTA, dehydrated in gradient ethanol, embedded in paraffin and then cut into 5 μm slices. The sections were stained with haematoxylin and eosin for general histology analyses.

### 2.11 Biomechanical testing

The femurs' mechanical properties with drill‐hole defects were examined at 2 and 4 weeks after surgery using a three‐point bending test. The intact contralateral femur was also tested as an internal control. A material test machine (Instron, 3343M1372) was used. The femurs were positioned horizontally with the anterior surface upwards and centred on the supports with 10 mm apart. The load was applied at the drill‐hole site regularly with a displacement rate of 5 mm/min and directed vertically to midshaft with anterior surface upward. After the femur was broken, the failure load and stiffness were recorded.

### 2.12 Immunofluorescence

To trace the cell fate of Prx1^+^ MSCs and Prx1^−^ MSCs, they were labelled with GFP using lentivirus (Cyagen Biosciences) and transplanted into the mice (n = 3 per group) with femoral bone defect. At 2 weeks after surgery, femurs were harvested, and fixed samples were decalcified, dehydrated and embedded in Tissue‐Tek^®^ OCT Compound (SAKURA). The 10 μm thickness of sagittal sections was cut with a freezing microtome (Thermo Scientific). The sections were blocked in 5% BSA for 40 minutes at room temperature and incubated with the primary antibodies anti‐DMP1 (1:400; Abcam) and anti‐GFP (1:400; Abcam) at 4°C overnight. After washing, the sections were then incubated with the respective secondary antibodies (1:500; Abcam) for 1 hour at room temperature and sealed with DAPI. The images were captured with a fluorescence microscope (Zeiss). For DMP1 quantification, Image J (1.52 version) was used to calculate the area percentage of DMP1 in the bone defect healing area.

### 2.13 Statistical analysis

All quantitative data were presented as mean ± standard deviation. ANOVA was conducted, followed by Bonferroni multiple comparison post hoc test for comparing variables among groups using GraphPad Prism 7 software. The differences were considered to be statistically significant when *P* < .05.

## 3 RESULTS

### 3.1 Prx1^+^ MSCs were involved in bone regeneration, and the numbers decreased with age

**FIGURE 1:**
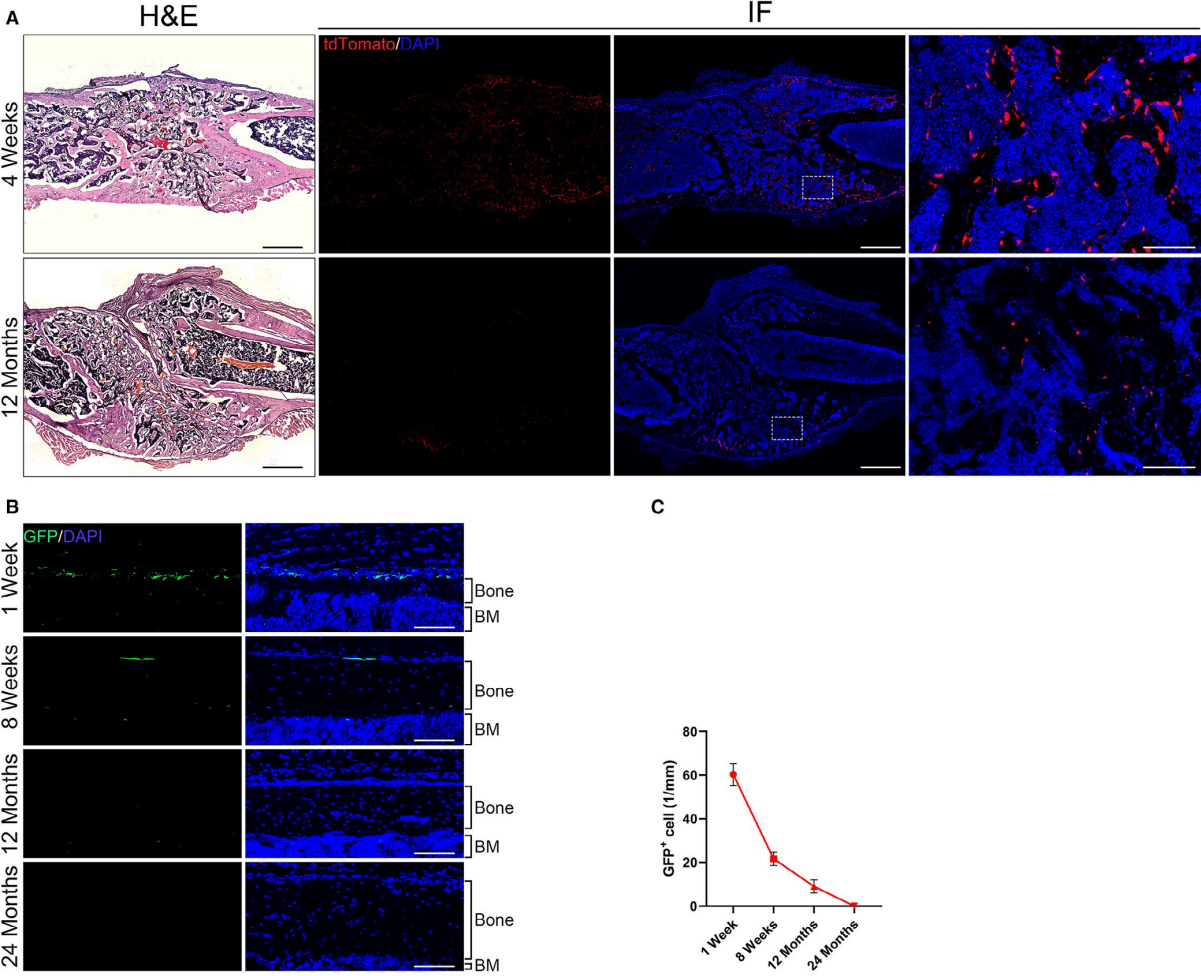
Prx1^+^ MSC was involved in bone regeneration, and the number decreased with age. A, Representative histological and immunofluorescence images of the femur at post‐operative 2 wk (scale bar: 500 mm). The white box showed the magnificent high resolution of the healing area (scale bar: 100 mm). B, Representative images of transgenic murine femurs at different age stained for GFP. GFP^+^ MSCs (green) were mainly localized within periosteum (scale bar: 100 mm). C, Quantification of GFP^+^ MSC at different age; BM: bone marrow. Data are presented at mean ± SD; *P < .05

To investigate whether Prx1^+^ MSCs involved in bone fracture healing in young and aged mice, 4 weeks and 12 months old Prx1CreER‐GFP;Rosa26^tdTomato^ mice were used to create femoral shaft bone fracture after 5 days tamoxifen injection. At 2 weeks post‐operatively, numerous tdTomato^+^ MSCs were presented in the newly formed bone in the femoral closed fracture model, indicating that Prx1^+^ MSCs were involved in bone regeneration and a pivotal cell population during fracture healing (Figure 1A and S1). At the same time, the number of tdTomato^+^ MSCs decreased significantly with age, which indicated that the involvement of Prx1^+^ MSC in bone regeneration decreased with age. To reveal the real‐time distribution of Prx1^+^ MSCs, 1 week, 8 weeks, 12 months and 24 months old Prx1CreER‐GFP mice were killed for analysis. Immunostaining of longitudinal periosteal sections of the femur of mice from early postnatal to late adulthood showed that GFP^+^ MSCs were abundant in the young murine periosteum and decreased markedly during late adulthood (Figure 1B,C). There were significant differences among them (*P* < .05 for all).

### 3.2 **Prx1^+^ MSC could be isolated from the periosteum and have the same proliferation ability as Prx1** ^−^ **MSC**

**FIGURE 2:**
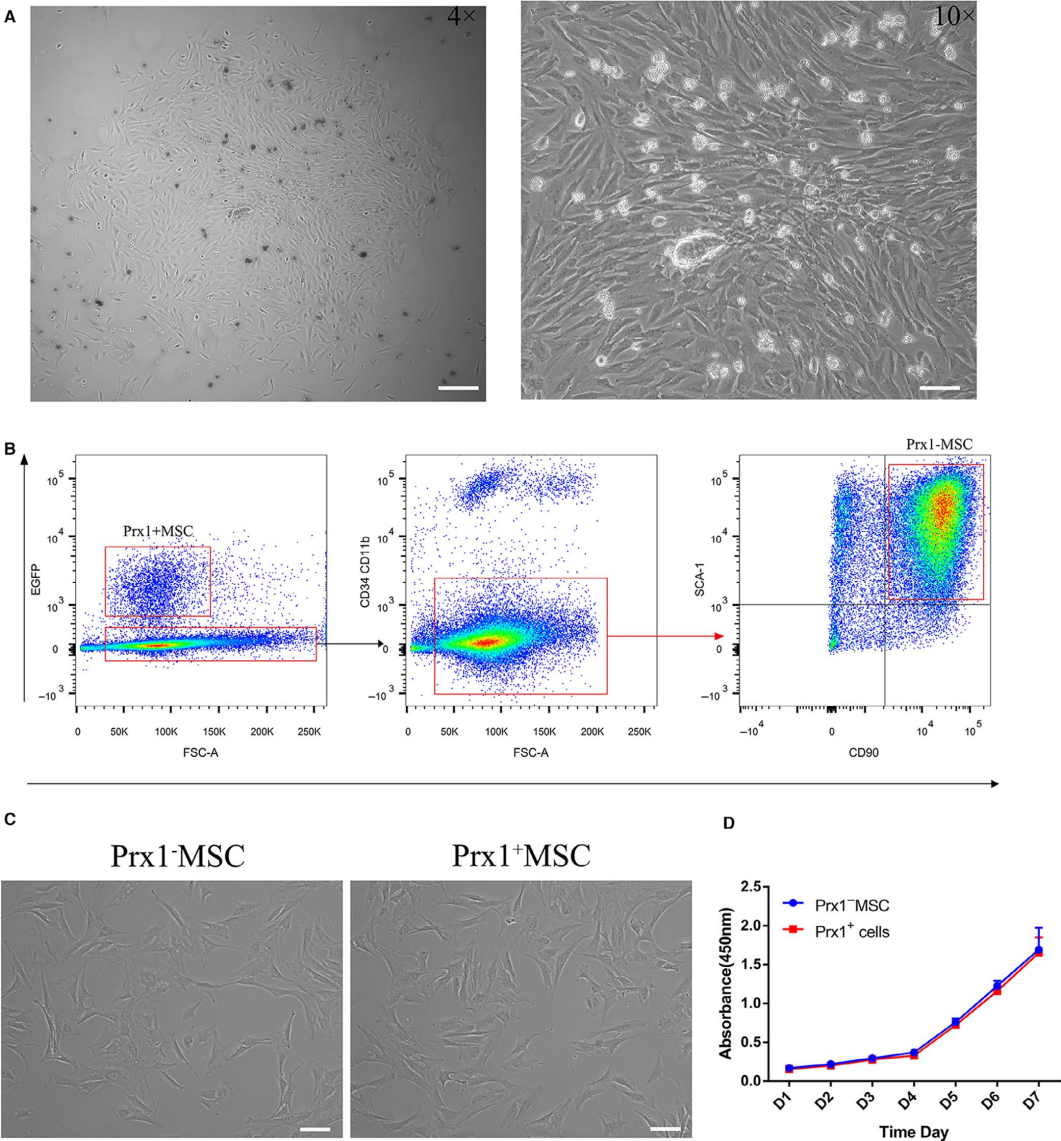
Isolating of the Prx1^+^ MSC and Prx1^−^MSC. A, Morphology of periosteum derived cells at passage 0 (Sale bar: 100 μm). B, Prx1^+^ MSCs and Prx1^−^ MSCs (GPF^−^CD90^+^Sca‐1^+^CD34^−^CD11b^−^ cells) were harvested by cell sorting. C, Representative image of Prx1^+^ MSC and Prx1^−^ MSC (Sale bar: 100 μm). D, Comparative cell proliferation assay of Prx1+ MSCs and Prx1‐ MSCs

After 3 days of culture, periosteal cells formed colony‐forming units (Figure 2A). Prx1^+^ MSCs and Prx1^−^ MSC were successfully sorted from murine limb (Figure 2B). They showed a spindle‐like morphology (Figure 2C) and had the same proliferation ability (Figure 2D).

### 3.3 **Prx1^+^ MSC showed higher osteogenic potency than Prx1** ^−^ **MSC**

**FIGURE 3:**
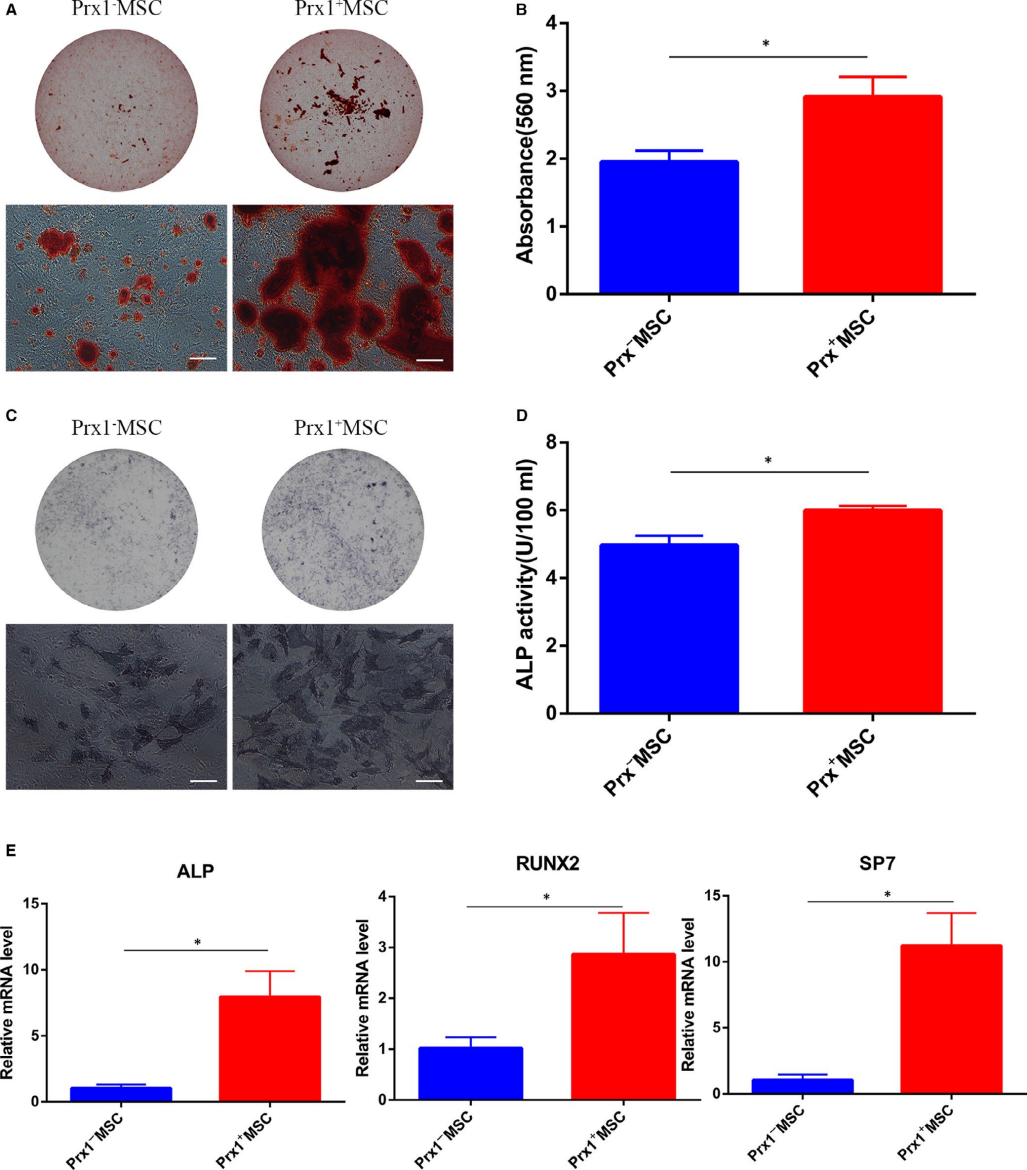
Comparison of osteogenic differentiation ability of Prx1^−^ MSC and Prx1^+^ MSC. A,, Gross view and representative image of Alizarin red staining of sorted cells induced in osteogenic medium for 3 wk (Scale bar: 100 μm). B, Quantitative analysis of Alizarin Red staining in A. n = 3 per group. C, Gross view and representative images of ALP staining after Prx1^+^ MSC and Prx1^−^ MSC induced in osteogenic medium for 7 d. Scale bar: 100 μm. D, Quantitative analysis of ALP activity. n = 3 per group. E, The osteogenesis‐related gene expression by Prx1^+^ MSCs and Prx1^−^ MSCs induced in osteogenic medium for 7 d. *P < .05

We then compared the osteogenic potency between Prx1^+^ MSC and Prx1^−^ MSC. Prx1^+^ MSC formed more calcium deposits compared with Prx1^−^ MSC, as based on Alizarin Red staining (Figure 3A). Accordingly, the quantitative analysis of Alizarin Red staining in Prx1^+^ MSC was remarkably higher than that in Prx1^−^ MSC (*P* < .05; Figure 3D). The osteogenic related gene showed higher expression in the Prx1^+^ MSC group than that in Prx1^−^ MSC group, and there was a significant difference between these two groups in ALP (*P* < .05), Runx2 (*P* < .05), SP7 (*P* < .05; Figure 3E).

### 3.4 Prx1^+^ MSC could promote early healing of aged murine bone defect

#### 3.4.1 Histology analyses

**FIGURE 4:**
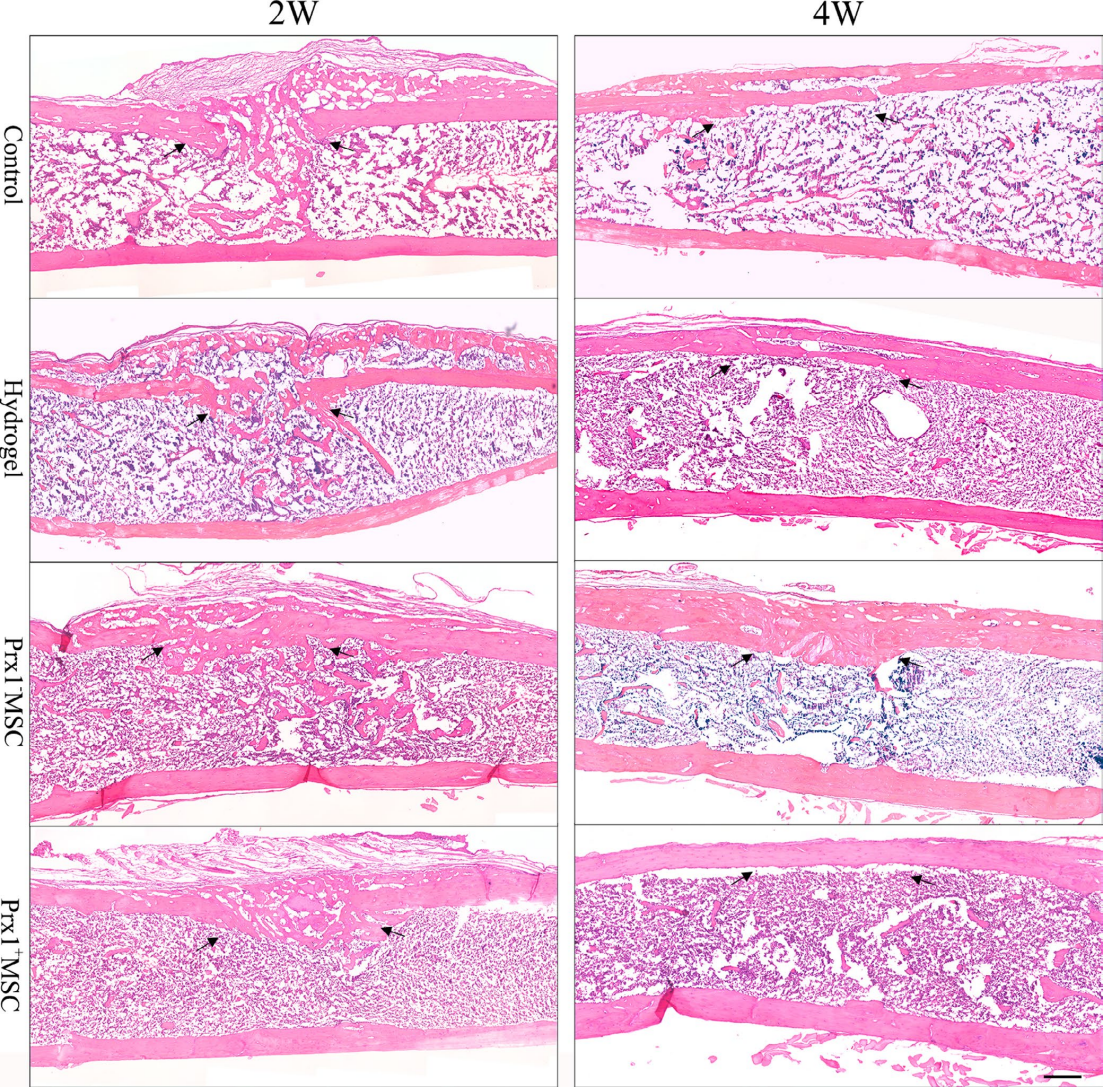
H&E staining analysis. Representative images of callus section in the sagittal view at post‐operative 2 and 4 wk. Arrows indicated the cortical gap (Scale bar: 200 μm)

At 2 weeks after surgery, H&E staining showed that the bone defect section was characterized by newly formed cancellous bone bridging the bone defect site in all groups. The Prx1^+^ MSC group and Prx1^−^ MSC group showed denser cancellous bone than the control group and hydrogel group in the cortical gap. Besides, the woven bone in the medullary cavity was largely absorbed in the Prx1^+^ MSC group. At 4 weeks after surgery, the bone defect in all groups was filled with dense woven bone with newly formed bone in the medullary cavity, which decreasing to near normal level. The cortical gap in the Prx1^+^ MSC group could not be identified, while the bone remodelling procedure in other groups was not yet completed (Figure 4).

#### 3.4.2 SR‐μCT analyses

**FIGURE 5:**
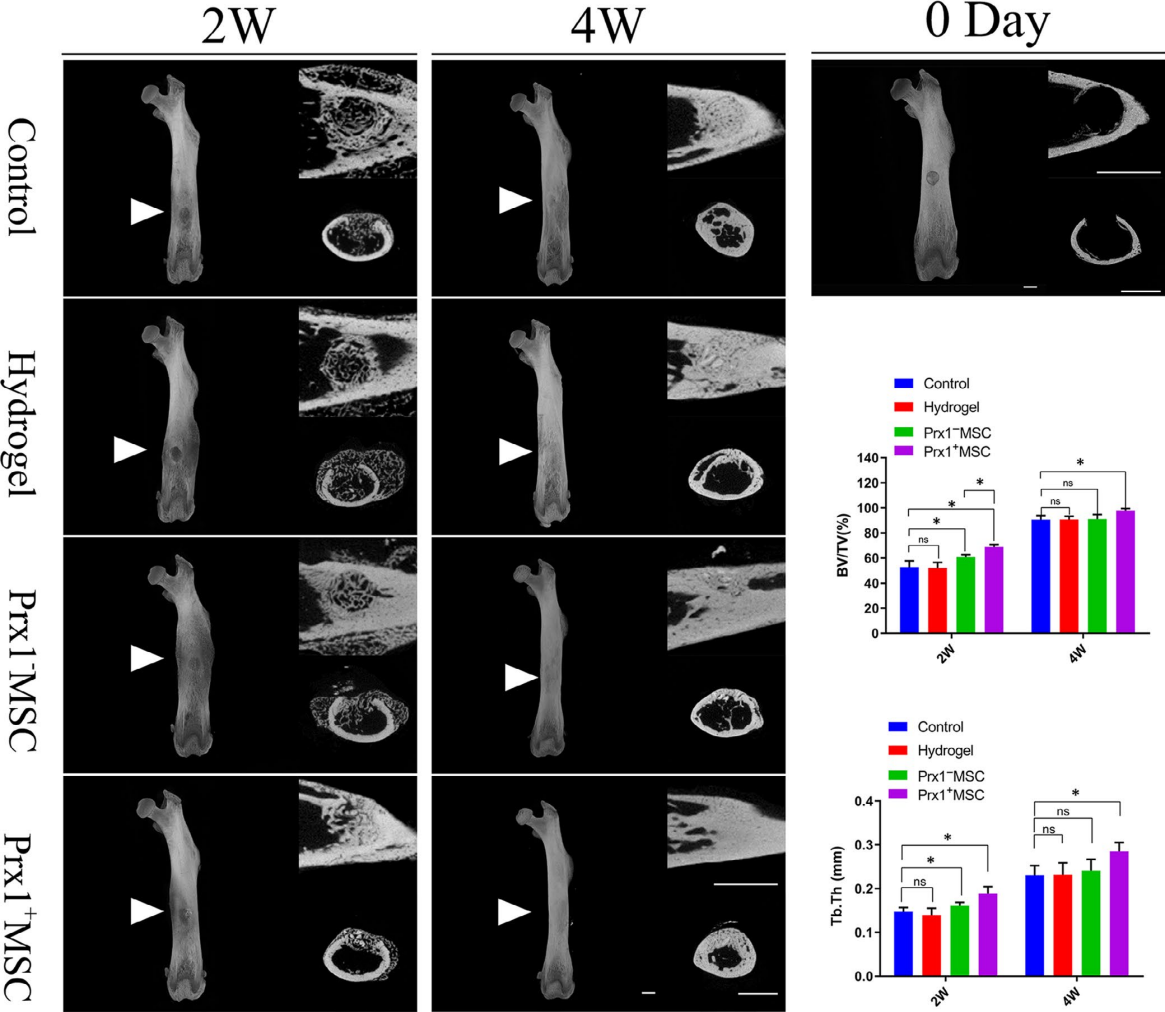
SR‐μCT analyses. A, Representative images of femurs in 3D view (the left side), coronal view (the above of right side) and horizontal view (the below of right side; Scale bar: 1 mm). B, Comparison of BV/TV in the bone defect area at post‐operative 2 and 4 wk. C, Comparison of Tb.Th in the bone defect area at post‐operative 2 and 4 wk. n = 5 per group. *P < .05

At 2 weeks after surgery, the bone defect in the Prx1^+^ MSC group and Prx1^−^ MSC group was vague, and newly formed trabecular bone beyond the defect area was mostly absorbed and remodelled in the Prx1^+^ MSC group. At 4 weeks after surgery, the newly formed bone had filled the defect area, which was hard to identify in all groups, while the remodelling of newly formed bone was nearly completed in the Prx1^+^ MSC group (Figure 5A). At 2 weeks after surgery, the Prx1^+^ MSC group showed a higher value of BV/TV and Tb.Th than other groups in the bone defect area. There were significant differences among them (*P* < .05 for all). Hydrogel alone did not improve the BV/TV and Tb.Th value, when compared with the control group (*P* > .05). At 4 weeks after surgery, the Prx1^+^ MSC group showed a higher value of BV/TV and Tb.Th in the bone defect area than other groups (*P* < .05 for all), and no significant differences were found among the rest groups (*P* > .05 for all; Figure 5B,C).

#### 3.4.3 Mechanical test

**FIGURE 6:**
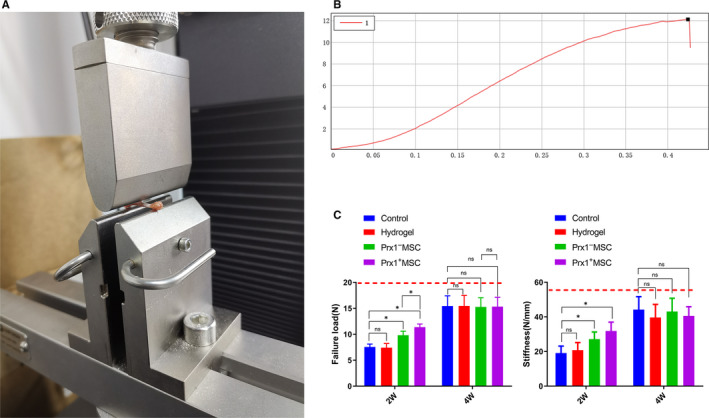
Mechanical test analysis. A, Gross view of the mechanical test machine. B, Representative load‐displacement curve. The failure load (C) and stiffness (D) of the femoral at post‐operative 2 and 4 wk. n = 6 per group. The red dotted line, respectively, indicated the mean failure load or stiffness of the uninjured murine femurs. *P < .05

During the mechanical testing, all samples were cracked at the defect part, and no one was excluded. At 2 weeks after surgery, the Prx1^+^ MSC group and Prx1^−^ MSC group exhibited a higher value of failure load when compared with the control group (*P* < .01 for all), but no significant difference was found between the hydrogel and control group (*P* > .05). The Prx1 + MSC group also showed a higher value of failure load than the Prx1^−^ MSC group. At the same time, the Prx1^+^ MSC group showed a higher value of stiffness than the control group (*P* < .01), but no significant difference was found among the other groups (*P* > .05 for all). At 4 weeks after surgery, failure load and stiffness in the four groups increased significantly, and no significant difference was found among them (*P* > .05 for all; Figure 6).

### 3.5 Prx1^+^ MSC could involve bone regeneration via intramembranous ossification

**FIGURE 7:**
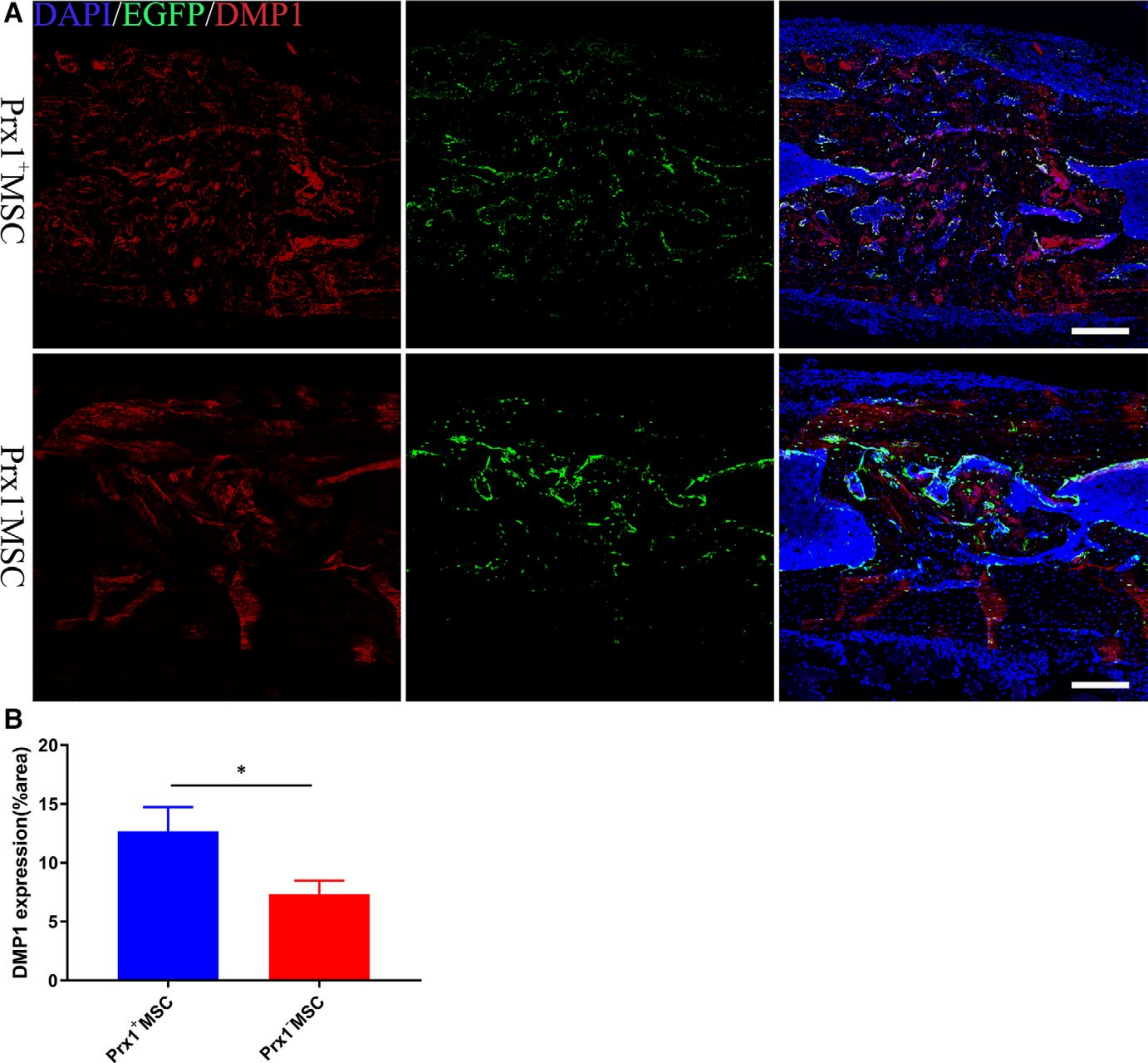
Periosteal stem cells improve bone regeneration via intramembranous ossification. A, Transplanted periosteal stem cells (green) could survive in the bone defect site and enhance bone regeneration via directly differentiating into osteoblasts. The mineralization degree, which indicated by DMP1 (red), was better in Prx1^+^ MSC group. B, Quantification of the DMP1 area percentage in the healing area. *P < .05

To investigate the fate of the transplanted cells, 12 months old mice were implanted with Prx1^+^ MSCs and Prx1^+^ MSCs, which were permanently labelled with GFP using lentivirus. Immunofluorescence showed that the transplanted periosteal stem cells could survive in the healing site and improve bone regeneration via directly differentiating into osteoblasts (Figure 7A). More dentin matrix protein 1 (DMP1) was found in the Prx1^+^ MSC group than in Prx1^−^ MSC group, indicating that Prx1^+^ MSC was better than Prx1^−^ MSC on enhancing bone regeneration in aged mice (Figure 7B). This effect was consistent with the result in vitro.

## 4 DISCUSSION

In this study, we found that Prx1^+^ MSC were mainly localized within the periosteum and highly participated in bone defect regeneration in young mice, while its number would decrease with age. Prx1^+^ MSC had better osteogenic differentiation potency than Prx1^−^ MSC. We transplanted these two cells into the bone defect site in aged mice. The results showed Prx1^+^ cells could significantly improve bone regeneration. Moreover, SR‐μCT exhibited high resolution and reliability in analysing bone regeneration.

Previous studies have mainly focused on the characterization of bone marrow‐derived mesenchymal stem cells,^25^ and adipose‐derived mesenchymal stem cells,^26^ which are currently used in cell‐based therapy approaches in orthopaedics for bone or cartilage regeneration. However, they found that endogenous BMSCs have a lower capacity to form cartilage and bone during skeletal regeneration than periosteum derived cells (PDCs)^21^ and primarily stimulate healing via the secretion of growth factors.^9^, ^27^ Therefore, more and more attention was paid to subpopulations of mesenchymal stem cells attributed to bone and cartilage progenitors.^28^ In our study, we focused on PDCs and tried to determine how the Prx1^+^ MSC, one population among them, take part in bone regeneration and whether impaired bone regeneration with ageing is associated with loss of Prx1^+^ MSC. The results showed that Prx1^+^ MSC could improve bone regeneration in aged murine bone defect model via direct cell replacement. Transplantation of Prx1^+^ cells into bone defect sites in aged mice would enhance the number of osteogenic progenitors in cells pooling, which could finally facilitate bone regeneration.

Periosteum derived cells are essential for bone regeneration.^9^, ^21^, ^29^ Many researchers have been trying to figure out the nature of the periosteum and subpopulations of PDCs.^15^, ^30^ There were reports showed that Nestin^+^ and LepR^+^ cells derived from periosteum were located in the outer layer of the periosteum surface, and they possessed self‐renewal capacity and committed to osteogenic lineage cells.^16^, ^31^ Unlike Prx1^+^ cells that were confined in the skeletal system, Nestin^+^ and LepR^+^ cells could also be found in other tissues.^32^, ^33^ Therefore, we thought the decreasing of Prx1^+^ cells with age could be strongly related to the impaired osteogenic potency in aged mice. Our results showed that Prx1^+^ MSC indeed could improve early healing of bone defects in aged mice. Meanwhile, Prx1^−^ MSCs could also improve bone regeneration, but this ability to promote bone regeneration was lower than that of Prx1^+^ MSC. In other words, there are different subpopulations of MSCs that take part in the bone healing. Prx1^+^ MSC is just one of them and maybe the most important one. Prx1^−^ MSCs may also contain subpopulations that play an essential role in bone regeneration or maintaining bone homeostasis, missing in aged mice that need further investigations. Still, we just focused on the change in the number of Prx1^+^ MSC without the potential change of differentiation and proliferation ability with age in this study. Further study is needed to investigate it.

According to a previous study, most of the newly formed bone in the marrow cavity will be absorbed, and newly formed bone in the cortical gap will be remodelled comparable into compact bone at post‐operative 2 weeks in young mice.^23^ Compared with this, the newly formed woven bone remodelling was incomplete in the control group with aged mice, as demonstrated by histology and SR‐μCT. The results indicated that bone regeneration ability was indeed impaired in aged mice. In general, senescence is defined by a minimum age of at least 18 months in mice.^34^ That is to say, the mice in the current study are not old. Because what we concerned most was if Prx1^+^ MSC could improve bone regeneration more than Prx1^−^ MSC in aged mice without Prx1^+^ cells. Twelve months old mice that were demonstrated to have no Prx1^+^ MSC could be used to test our experimental hypotheses to some extent.

In this study, a single femoral bone defect (0.8 mm in diameter) was made in the midshaft. As we known, the bone fracture was an ideal model to simulate clinical patients bone regeneration situation.^35^ But we could not avoid the bias brought by stabilization intervention, which could influence the result that Prx1^+^ cells can improve the bone regeneration. Meanwhile, this model had been proved that it was a valid and reliable model for the evaluation of bone regeneration. It had the following advantages. The defect did not need stabilization devices and did not result in a high incidence of fracture. However, we found no significant difference in mechanical test results between all groups at post‐operative 4 weeks, although Prx1^+^ MSC group had better radiographic results than other groups. To some extent, the time‐point of 4 weeks after surgery was not enough for assessing the mechanical test results in this model. More time‐points are needed in future work.

Still, there were some limitations to the current study. First, we transplanted the passage three cells to the defect site, which might cause some changes in the PDCs' identities in vitro cultivation. Second, for Prx1^+^ MSC tracing, tamoxifen was used here to induce the tdTomato expression to label the Prx1^+^ MSC permanently. We did not rule out the potential influence of tamoxifen on the Prx1^+^ MSC participating bone regeneration. Third, our sample size was too small. Large sample size experiments using large animals as models should be conducted for further investigation.

## 5 CONCLUSION

In conclusion, transplanting PDCs into the bone defect site in 12 months old mice could stimulate bone regeneration. The decreased bone regeneration ability in aged mice might be related to the dropping of Prx1^+^ MSC number within the periosteum.

## CONFLICT OF INTEREST

The authors declare no competing financial interests.

## AUTHOR CONTRIBUTIONS

**Han Xiao:** Conceptualization (lead); Data curation (lead); Investigation (lead); Methodology (lead); Writing‐original draft (lead); Writing‐review & editing (lead). **Linfeng Wang:** Conceptualization (lead); Data curation (lead); Investigation (lead); Methodology (lead); Writing‐original draft (lead); Writing‐review & editing (lead). **Tao Zhang:** Formal analysis (supporting); Software (supporting). **Can Chen:** Formal analysis (supporting); Supervision (equal). **Huabin Chen:** Methodology (supporting); Software (supporting). **Shengcan Li:** Formal analysis (supporting); Investigation (supporting). **Jianzhong Hu:** Conceptualization (lead); Funding acquisition (lead); Project administration (lead); Validation (lead). **Hongbin Lu:** Conceptualization (lead); Data curation (lead); Funding acquisition (lead); Project administration (supporting); Supervision (lead); Validation (lead); Writing‐review & editing (lead).

## Supporting information

Fig S1

## Data Availability

The data that support the findings of this study are available on request from the corresponding author. The data are not publicly available due to privacy or ethical restrictions.
