# Supplementary material for: Periosteum progenitors could stimulate bone regeneration in aged murine bone defect model
Source: J Cell Mol Med. 2020 Sep 15;24(20):12199–210. doi: 10.1111/jcmm.15891 (PMC7579685; doi:10.1111/jcmm.15891)
Supplement: Supplementary file 1 — Fig S1 [file JCMM-24-12199-s001.docx]

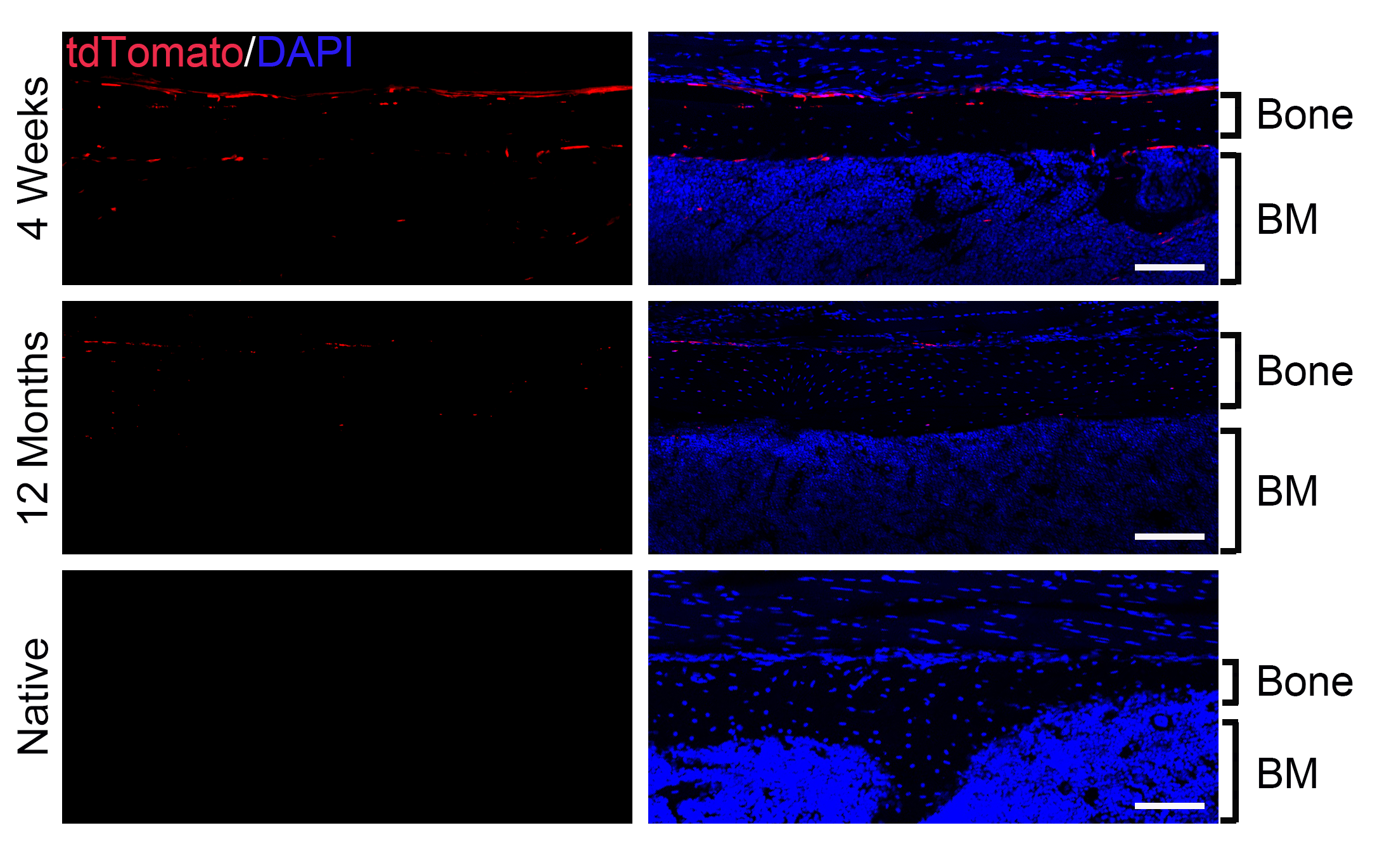


Figure S1: Representative immunofluorescence images of the femur in 4 weeks and 12 months old mice which were injected with tamoxifen for 5 days. Native refers to the 4 weeks old mice without injection of tamoxifen (Scale bar: 100μm). BM: bone marrow.
